# Evaluation of Tissue Interactions with Mechanical Elements of a Transscleral Drug Delivery Device

**DOI:** 10.3390/pharmaceutics4010212

**Published:** 2012-03-12

**Authors:** Sarah J. Cohen, Robison V. Paul Chan, Mark Keegan, Christopher M. Andreoli, Jeffrey T. Borenstein, Joan W. Miller, Evangelos S. Gragoudas

**Affiliations:** 1 Charles Stark Draper Laboratory, 555 Technology Square, Cambridge, MA 02139, USA; Email: sjcohen@alum.mit.edu (S.J.C.); mek10@cornell.edu (M.K.); 2 Massachusetts Eye and Ear Infirmary, 243 Charles Street, Boston, MA 02114, USA; Email: roc9013@med.cornell.edu (R.V.P.C.); christopher_andreoli@meei.harvard.edu (C.M.A.); joan_miller@meei.harvard.edu (J.W.M.); evangelos_gragoudas@meei.harvard.edu (E.S.G.)

**Keywords:** age-related macular degeneration, drug delivery, transscleral

## Abstract

The goal of this work was to evaluate tissue-device interactions due to implantation of a mechanically operated drug delivery system onto the posterior sclera. Two test devices were designed and fabricated to model elements of the drug delivery device—one containing a free-spinning ball bearing and the other encasing two articulating gears. Openings in the base of test devices modeled ports for drug passage from device to sclera. Porous poly(tetrafluoroethylene) (PTFE) membranes were attached to half of the gear devices to minimize tissue ingrowth through these ports. Test devices were sutured onto rabbit eyes for 10 weeks. Tissue-device interactions were evaluated histologically and mechanically after removal to determine effects on device function and changes in surrounding tissue. Test devices were generally well-tolerated during residence in the animal. All devices encouraged fibrous tissue formation between the sclera and the device, fibrous tissue encapsulation and invasion around the device, and inflammation of the conjunctiva. Gear devices encouraged significantly greater inflammation in all cases and a larger rate of tissue ingrowth. PTFE membranes prevented tissue invasion through the covered drug ports, though tissue migrated in through other smaller openings. The torque required to turn the mechanical elements increased over 1000 times for gear devices, but only on the order of 100 times for membrane-covered gear devices and less than 100 times for ball bearing devices. Maintaining a lower device profile, minimizing microscale motion on the eye surface and covering drug ports with a porous membrane may minimize inflammation, decreasing the risk of damage to surrounding tissues and minimizing disruption of device operation.

## 1. Introduction

A variety of diseases affect the posterior segment of the eye, including age-related macular degeneration and diabetic retinopathy. Drug treatments have shown promise for these diseases [1,2,3,4,5], but delivery of these agents to the posterior segment over long time periods remains a challenge. The current widely used method of intra-ocular injection delivers the drugs locally, significantly limiting any systemic side effects. However, limited injection volumes and half-lives in the vitreous mandate periodic repeat injections, which are inconvenient for the patient and result in recurring risks of cataract formation or progression [6,7], endophthalmitis [8] hemorrhage and retinal detachment [9]. 

As an alternative to intraocular injections, trans-scleral diffusion of drug has been explored as potential route for delivery [10]. Studies have shown the sclera to be permeable to molecules including betamethasone [11,12,13], oligonucleotides [14,15], albumin [16,17] and the anti-angiogenic molecules carboxyamido-triazole and 2-methoxyestradiol [18]. These molecules have ranged up to 150 kDa in molecular weight in situations of passive diffusion [19], osmotic pumping [20], and elevated intraocular pressure [17].

Delivery of drug to the posterior segment of the eye presents significant clinical challenges. Potential methods for achieving this delivery route include drug loaded polymers and iontophoresis. Drug loaded polymers have been implanted in disc form intra-sclerally [12] and injected in microspheres periocularly [15,21,22]. The latter technique has enabled drug encapsulation in biodegradable nanospheres to transport drug to ocular tissues for sustained release [21]. These particles range 200–500 nm in diameter and sustain the release of drug longer than injection of drug in solution [23,24,25]. Iontophoresis has been investigated as a method of electrically driving transport of drug across the sclera [26,27]. While these methods may prove useful for short-term, sustained delivery, they do not allow for long-term, programmable delivery of therapy. In response to the need for miniaturized long-term implantable devices, refillable micro-electrical mechanical systems (MEMS) devices are currently being developed to pump drug solution through a cannula inserted in the sclera [28,29,30,31]. Related MEMS-based micropumps are also in development for intracochlear drug delivery [32]. 

**Figure 1:**
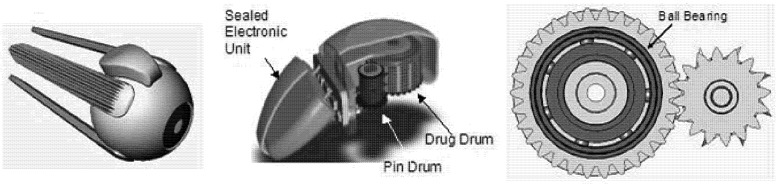
Transscleral drug delivery device. The device is designed to reside on the posterior sclera (left) and contains two drums that rotate against each other via gear mechanism (cutaway view, middle). The protruding blades on the pin drum periodically puncture wells on the drug drum as they rotate. A ball bearing may also be used to support the rotational movement of the drug drum (bottom view of drums, right).

Along these lines, we have previously proposed a sclera device storing drug solids that would deliver the drug to the interstitial fluid for passive diffusion across to the retina and choroid [33,34] (Figure 1). This device would be mechanically operated, containing components that sequentially release a dose every 3–6 days for as long as two years. This platform could support a variety of drugs, including multiple drugs within a single device, with a total storage volume of 30 microliters. Small molecules capable of inhibiting neovascularization in the choroid may be a good candidate for use in this device; the drug may be stored dry and then released into the fluid-filled space above the eye during delivery. Due to its magnetically controlled actuation method, there is also the possibility of programming patient-specific dosing scenarios. While the battery and electronic elements could be sealed inside the device, in order to deliver drug, the internal mechanical components may require exposure to the surrounding tissues.

As an implanted foreign body, this device has the potential to erode, tear or otherwise damage surrounding tissues. It could also initiate an inflammatory response that leads to fibrous encapsulation, potentially limiting eye motion or mechanically restricting rotation of functional components inside the device [35,36,37]. Of the two devices commonly implanted on the sclera, both scleral buckle explants and glaucoma drainage devices (GDDs) affect surrounding tissues and encourage a degree of inflammation. Many silicone scleral buckle materials have been designed to be mechanically pliable, so as to minimize erosion to the sclera during long-term attachment. These devices do encourage formation of a surrounding fibrous capsule, though for this static, monolithic device, the capsule has the advantages of securing the buckle material and strengthening the sclera [38,39]. For GDDs, similar fibrous encapsulation around the scleral plate may occlude the drainage port on the device and is the leading cause of device failure. Implantation of the plate alone results in a layer of avascular fibrous tissue around the material that remains consistently after 4 weeks, resulting in varying degrees of inflammation with different materials [40]. 

Due to the potential for these complications, understanding the local physiological effects of implanting the proposed drug delivery device onto the sclera for long periods is desirable. The focus of the current work was to identify design criteria that would reduce the overall likelihood of local tissue damage and scar tissue formation, in the absence of anti-inflammatory drugs or other means to suppress foreign body response. Here we describe results from an *in vivo* rabbit model to investigate the feasibility of implanting this type of drug delivery device by suturing to the posterior sclera. 

## 2. Experimental Section

### 2.1. Test Device Development

The drug delivery device was designed to contain two cylindrical drums that rotate against each other. The “drug drum” contains compartments of drug, and the “pin drum” supports blades that periodically puncture the seal on each compartment (Figure 1). The device uses a set of rotating mechanical elements to support the drum motion, including both gears and ball bearings. The casing that encloses these elements is rounded to mate with the eye wall and contains drug ports that enable drug diffusion. Because of the intricacy and durability required for the elements of the device, the proposed design is most compatible with fabrication by machining of metallic materials [33,34]. 

To simplify *in vivo* experimentation, two test devices were designed to represent key elements of the drug delivery system, one containing a set of gears and one housing a ball bearing. Their design allowed for mechanical evaluation of the moving elements and histological evaluation of surrounding tissues. Each device was constructed entirely from biocompatible stainless steel (AISI 316L) or titanium alloy (Ti6Al4V). Because the proposed drug delivery device turns its gears slowly (approximately 15° of rotation per day if dosing every three days), it was proposed that a non-powered, static test device would model a reasonable worst-case scenario for degradation of system performance after residence in the body. 

Both devices were designed to be machined with rectangular base plates, spherically rounded and highly polished on one side to mate with the globe. All other external faces had low surface roughness. Internal surfaces of the gear box and ball bearing devices were fabricated with a uniformly textured surface to support adhesive bonding. Devices also included suture tabs protruding from two sides of the device as well as long slots cut through the thickness of the baseplate to allow for fluid communication into the device. Moving elements had smooth surfaces and were seated on the flat side of the baseplate, held in place with a device cover or set of covers. Gear device covers were designed to rest over the device without bonding to the gearbox, resulting in a gap between the cover and gearbox. 

It was hypothesized that covering the baseplate slots with a porous material may prevent cell migration into the device while still allowing the potential for drug passage out of the device. To investigate this possibility, an 80 micron thick poly(tetrafluoroethylene) (PTFE) membrane with 10 micron pores and a hydrophilic surface treatment (OmniporeTM, Millipore, Billerica, MA) was applied to the curved base of a subset of gear devices. This material was chosen based on the following criteria: material biocompatibility, pore sizes on the order of a cell diameter (10 µm), thickness less than 100 µm to ensure proper fit and the ability to prevent cell migration through its thickness. Pilot *in vitro* experiments in our lab confirmed that fibrous cells attached to one side of this material did not migrate through to the opposite side [41]. 

**Table 1:** Devices in each experimental group.

Group Name	Device Type	Materials	Number of Devices
G	Gear	Stainless steel (AISI 316L)	4
GM	Gear + PTFE Membrane	Stainless steel (AISI 316L), PTFE	4
BB	Ball Bearing	Titanium (Ti6Al4V), Zirconia	3

### 2.2. *In Vivo* Study

Fibrous encapsulation of a foreign body is known to occur within three weeks after implanting in the body [36]. Noting experimental logistics and referencing other studies [40,42,43], it was determined that a 10 week device residence time would sufficiently show the comparative effects of long-term residence in the body. 

Rabbit models have been used often for studies of ophthalmology, including evaluations of inflammatory response around a scleral device [40,42]. While differences in ocular inflammatory response may exist between the species, the rabbit and human conjunctival mucosa immune systems have several similarities [44,45]. The rabbit model was chosen as an appropriate model to provide insight into trends in the ocular foreign body response. 

Long term *in vivo* effects were investigated 10 weeks after implantation on a rabbit eye. Three types of devices were evaluated (Table 1): stainless steel gear devices (G), stainless steel gear devices with PTFE membranes (GM), and titanium ball bearing devices (BB).

Animals for this experiment were 5 months old at the time of implant. The Massachusetts Eye and Ear Infirmary Animal Care Committee approved this protocol, which adheres to the ARVO Statement for the Use of Animals in Ophthalmic and Vision Research. Before implantation, devices were disinfected for 2 hours in a 70% ethanol solution and stored in sterile bags until use. While this manner of sterilization would not be recommended for clinical use, others have used similar techniques for *in vivo* implantation in animals [42,47]. 

Animals were anesthetized with 1–1.5 mL of a 1:1 mixture of 50 mg/ml ketamine (Phoenix Pharmaceutical, St. Joseph, MO) and 20 mg/ml xylazine (Phoenix Pharmaceutical) by intramuscular injection. Only one device was attached per eye. For each eye receiving a device, the lids were separated and held open by a speculum. Eyes were prepared and anesthetized with betadine and proparacaine (Alcon, Fort Worth, TX). The conjunctiva and Tenon’s capsule were dissected from the globe in the area between the superior and lateral rectus muscles. Two stitches were placed in the sclera with 7-0 nylon suture (Ethicon, Somerville, NJ) and threaded through the suture holes on the device. Sutures secured the device on the globe with a 1-1-1 knot on either side. For the gear device, sutures were placed approximately 4.5 mm posterior to the limbus, with 9 mm between the two. For the ball bearing device, sutures were placed approximately 6 mm from the limbus, with 7 mm between the two. This configuration allowed at least 2 mm between the limbus and the device edge.

The conjunctiva was pulled back over the device and secured with a 2-1-1 knot of 7-0 Biosorb sutures (Alcon, Fort Worth, TX). Antibiotic drops and ointment were applied, and the speculum was removed. For the first week post-surgery, animals were monitored daily and antibiotic ointment was applied to prevent infection due to the surgical procedure. Animals were monitored weekly for the remainder of the study. 

Ten weeks after implant surgery, the animals were anesthetized with 1–1.5 mL of a mixture of 50 mg/mL ketamine (Phoenix Pharmaceutical) and 20 mg/mL xylazine (Phoenix Pharmaceutical) by intramuscular injection. They were euthanized by an intraperintoneal or intracardial overdose of pentobarbital sodium (Vortech Pharmaceutical, Dearborn, MI). Each eye was enucleated, taking care to leave any tissue surrounding the device intact. Without removing this tissue, devices were cut away from the scleral surface as needed to disengage the device from the eye.

Immediately after removal from the animal, devices were stored at 4°C in a tube containing a saline-soaked wipe to preserve the tissue before testing. Gross observations were photographed of the intact devices before mechanical testing and of dissected devices after testing. Tissue surrounding and permeating the device was removed and fixed for histology in either 10% formalin (Electron Microscopy Sciences, Fort Washington, PA) or in Karnovsky’s fixative (Electron Microscopy Sciences). Devices were thoroughly disinfected and cleaned after use, and some gearbox parts were reused for future tests. Reused gearbox parts were tracked and evenly distributed throughout the groups so as not to bias results. 

### 2.3. Mechanical Evaluation

**Figure 2:**
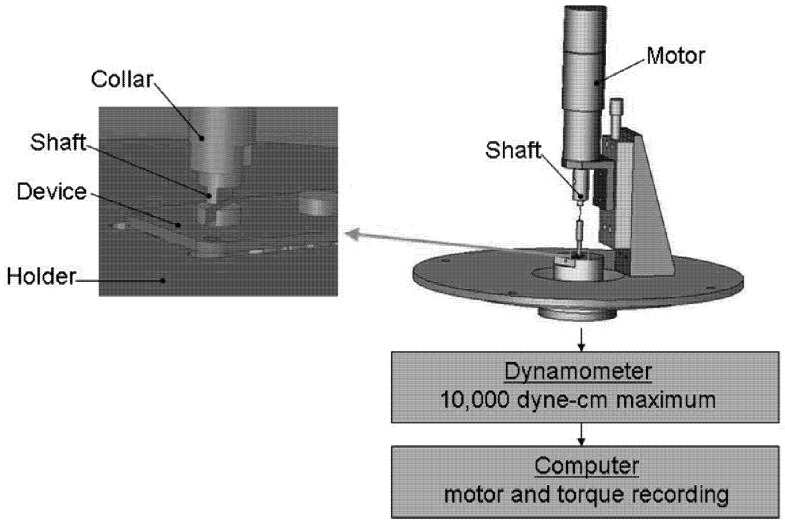
Mechanical testing apparatus. The apparatus consisted of a driving motor that was sequentially coupled to the rotating elements of each device. Devices were held in place on a dynamometer by a custom designed support, and torque required to turn the moving elements was recorded digitally.

The effect of residence *in vivo* on device function was measured by the amount of torque necessary to turn the moving elements. Each device was tested both before and after implantation on a dynamometer apparatus (Dynamics Research Corporation, Newton, MA, Model 4361, Mod 5) modified with a device holder and a motor coupled to the moving elements of the device (Figure 2). As constant voltage (215 mV, 275 mV and 400 mV for each device) was applied to the motor, allowing it to turn the gears or ball bearing, the dynamometer measured the amount of torque required of the motor to turn the elements. 

Torque readings were recorded over 10 rotations using a LabView (National Instruments, Austin, TX) computer interface. In some cases, the dynamometer apparatus could not supply sufficient torque, and measurements were taken by manually rotating the device elements with a torque gauge. An average, steady state value was calculated from this data for each test and was used for all statistical comparisons. To investigate differences between experimental groups, a normalized torque value was calculated for each device as the ratio of its post-explantation torque to its pre-implantation torque. A normalized torque value of 2 would then indicate that twice as much torque was required after explant. The data was organized by experimental group and applied to one-tailed Student’s t-tests.

## 3. Results and Discussion

### 3.1. Device Design

**Figure 3:**
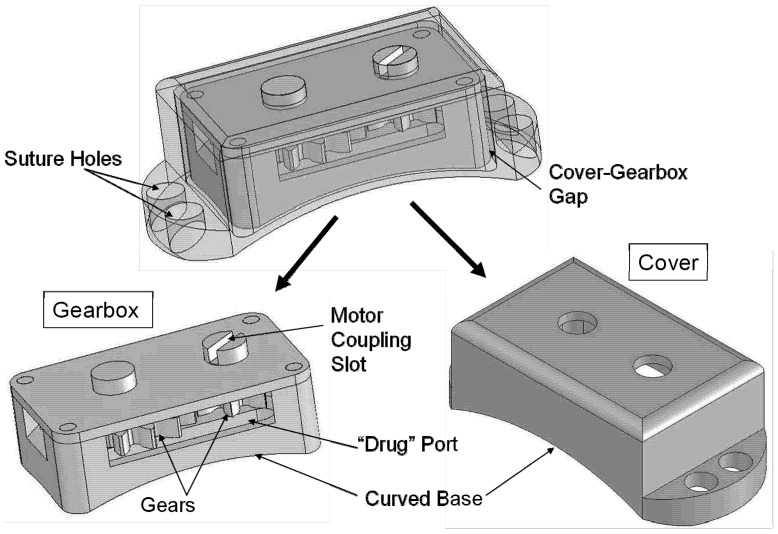
Overview of the Gear device. Overall dimensions: 3.05 mm (height) × 4.47 mm (width) × 9.43 mm (length). The device consists of a gearbox containing two articulating gears, plus a device cover that protects the gearbox and secures it to the sclera.

**Figure 4:**
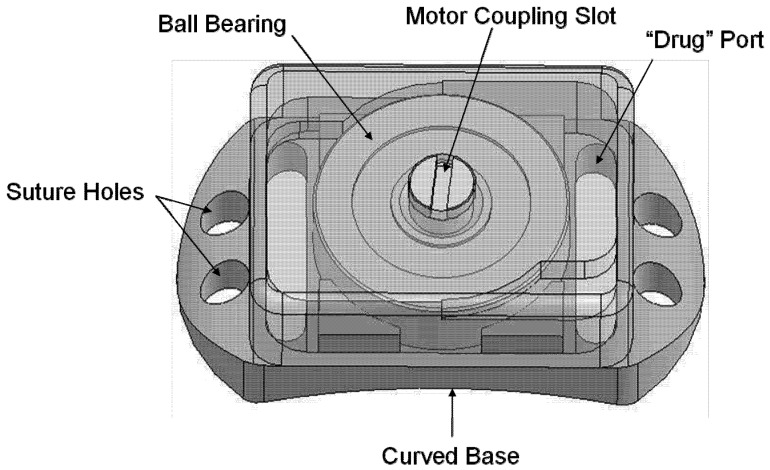
Overview of Ball Bearing device. Overall dimensions: 2.90 mm (height) × 4.57 mm (width) × 8.22 mm (length). The device consists of a baseplate supporting one ball bearing with a device cover bonded around its edges to the baseplate.

**Figure 5:**
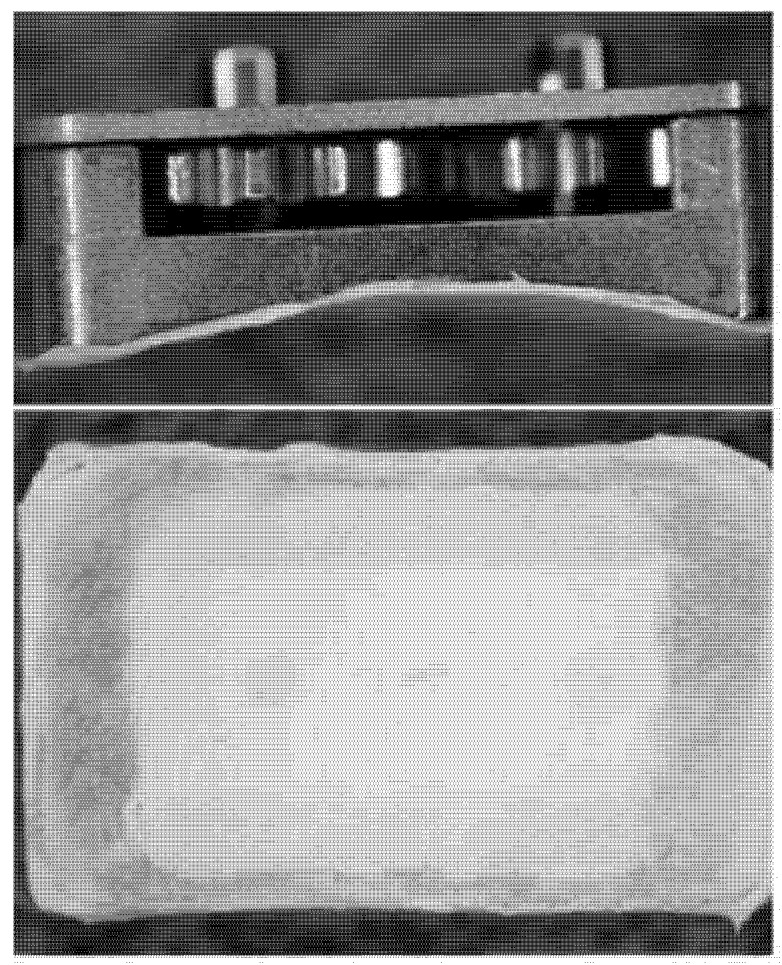
Photograph of a gearbox with a porous poly(tetrafluoroethylene) (PTFE) membrane covering the curved base. Membranes were applied with adhesive around the edges of the part. Membrane material beneath the drug ports was free from adhesive.

The drug delivery device is described in Figure 1, and the two types of test devices are shown in Figure 3 and Figure 4 below. An example gear device with membrane covering is show in Figure 5. 

### 3.2. Gross Observation

Animals tolerated the devices well. Within one week after implant, any redness or swelling around the eye had decreased significantly. There was no evidence of local tissue erosion in the area. 

A set of sutures had come loose from one of the ball bearing devices during the final week of residence in the body, allowing the device to move freely in front of the rabbit’s cornea. Infection was evident inside the device and in the animal’s cornea, but not in the tissues near the conjunctiva. Since this was the only device that presented in this way, it is suspected that the infection developed due to the external exposure after the device became dislodged. The device was otherwise evaluated in the same manner as the rest of the group.

### 3.3. Conjunctiva Reaction

Around all gear devices, the conjunctiva was significantly thicker than normal after the 10-week device residence period, often tough and somewhat gelatinous in texture. Ball bearing devices induced less fibrosis in the conjunctiva, but were also shrouded in a thin, distinct, relatively transparent tissue. 

**Figure 6:**
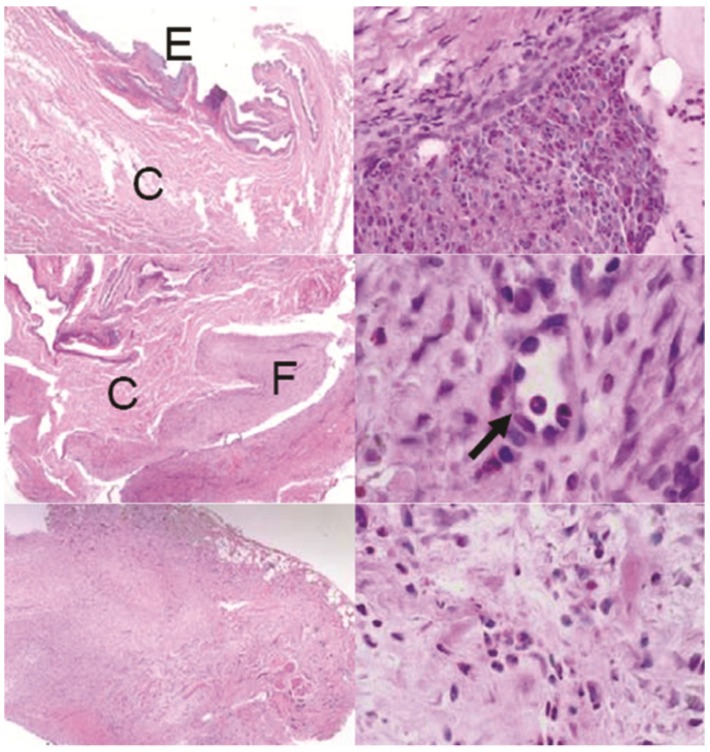
Sections of the conjunctiva above devices: Top left: control animal with no device showing the epithelial layer (E) and connective tissue (C), top right: one GM device with invasive fibrosis, middle row: one BB device showing fibrosis (F) and neovascularization (arrow), bottom row: one G device showing excessive fibrosis disrupting the conjunctiva architechture. H&E stain. Left row: all images 40×, right top: 400×, right middle: 1000×, right bottom 600×.

 Histological sections through the conjunctiva showed invasion by new fibrous tissue in the stromal layer for all devices (Figure 6). Ball bearing devices showed less fibrosis than the gear devices. The fibrous tissue disrupted the entire architecture of the conjunctiva in the gear device case, as compared to the ball bearing group, where the fibrosis grew as an extra layer inside the stroma. For most devices, inflammatory granulocytes occupied small focal areas within this tissue. Neovascularization in the newly formed fibrous tissue was evident in several samples. 

While a foreign body response was observed around all devices, the degree of scarring in the conjunctiva was much greater with the gear device than with the ball bearing device. We hypothesized that the outer shape of the device was most likely responsible for the difference between these two groups. The gear devices were narrower and taller than the ball bearing devices due to design constraints. They therefore disrupted natural eye lid movement to a greater degree and created a greater “dead space” between the globe and conjunctiva [48,49].

### 3.4. Eye Wall Reaction

**Figure 7:**
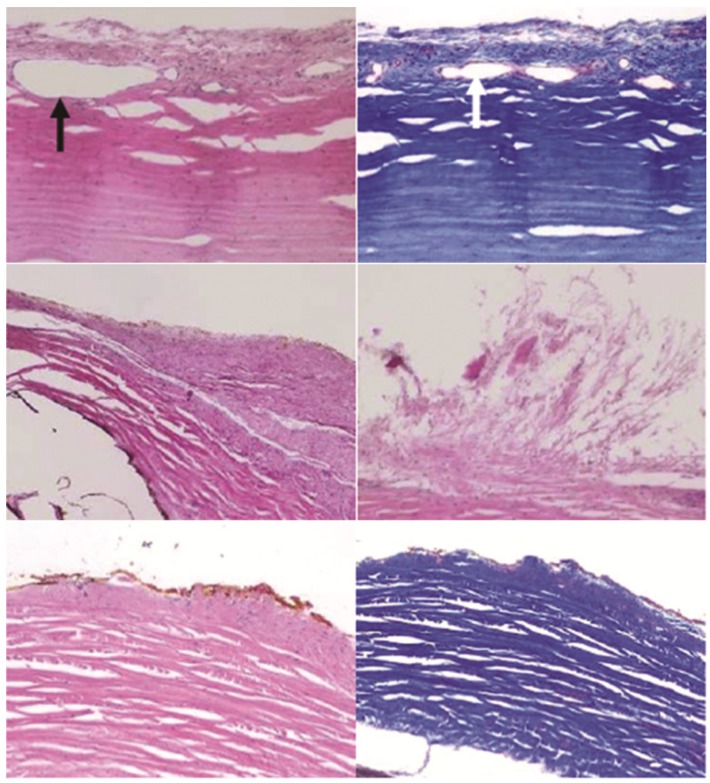
Sections of the sclera beneath devices. Images indicate a layer of fibrous tissue growth on the exterior of the sclera for all devices. Arrows indicate neovascularization. Top row: one BB device, middle row: two G devices, bottom row: one GM device. Left column and middle right: H&E stain, Top and bottom right: trichrome, all 100× magnification.

Eye wall histological sections showed no erosion to the sclera itself. On the contrary, all devices encouraged a layer of new growth continuous with the sclera in the area between the device and the eye (Figure 7). This new tissue acted to hold the device tightly in place on the eye. 

Based on histological analysis, this tissue was densely fibrous and included mostly fibroblasts, with occasional inflammatory cells. The new tissue disrupted the architecture of nearby muscles. Some neovascularization was apparent in this tissue, as noted in the ball bearing group. No consistent differences were identified between the scleral surfaces of experimental groups. While there were differences in thickness of the fibrosis between samples, it was not conclusively correlated to any particular device type.

New tissue growth on the sclera did not appear to harm the tissue, but it could alter the diffusion profiles of the drug to the retina. Transscleral drug diffusion studies have previously been conducted with healthy sclera [11,12,13,14,15,16,17,18,19,20]. The added tissue thickness and neovascularization could decrease the ability of a drug to diffuse through it. Future permeability studies are necessary to address the efficacy of drug delivery in the presence of added tissue. Device design and surgical implant procedure development may also be considered in order to minimize the thickness and effect of this fibrous layer. 

### 3.5. Tissue Ingrowth

**Figure 8:**
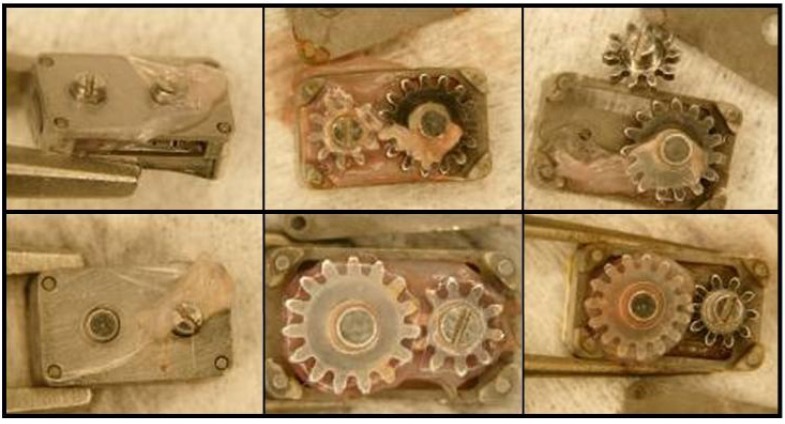
Tissue growth in Gear devices. Devices without membranes (top row) encouraged a relatively disorganized fibrous tissue growth around the gears. Devices with PTFE membranes (bottom row) encouraged a sac of fibrous tissue to form around the gear train inside the device. A thin layer of tissue was found surrounding all gear boxes (left column).

Gross inspection of device interiors revealed tissue ingrowth in most cases. Beneath their covers, all gearboxes (both with and without PTFE membrane) were encased in a thin, smooth, translucent layer of tissue (Figure 8), and the interior space was filled with a tougher, less organized tissue. The PTFE membranes appeared to prevent tissue growth through the drug ports; any tissue that grew into the drug ports from inside the device was not attached to the membrane. Additionally, tissue in the gear space of membrane devices tended to encase one or both gears into a single sac, while in uncovered devices, tissue tended to fill any open space. 

**Figure 9:**
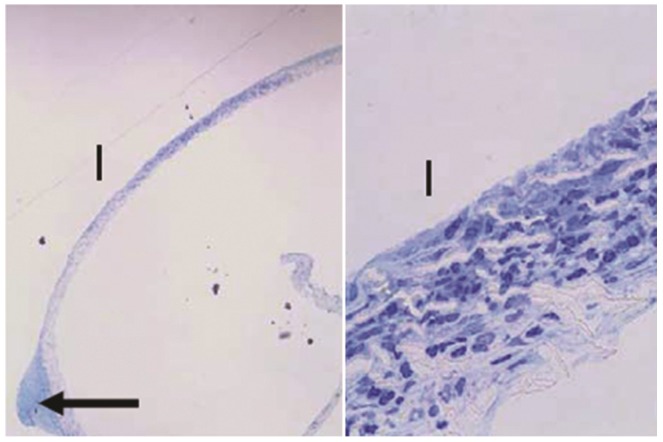
Sections of the PTFE membrane from the base of one GM device, where "I" denotes the internal side of the membrane facing the device. The arrow indicates bulb of tissue protruding into an opening in the baseplate. Toluidine blue stain. Left: 40×, right: 600×.

Sections through the PTFE membranes from gear devices revealed two bulbs of tissue protruding into the gear shaft holes of the device baseplate (Figure 9). Stemming from those regions, cells were present throughout the thickness of the membrane in the mid-section of the filter. The bulbs of tissue were not connected to any other internal material, suggesting that the cells had migrated through the membrane from the scleral side.

**Figure 10:**
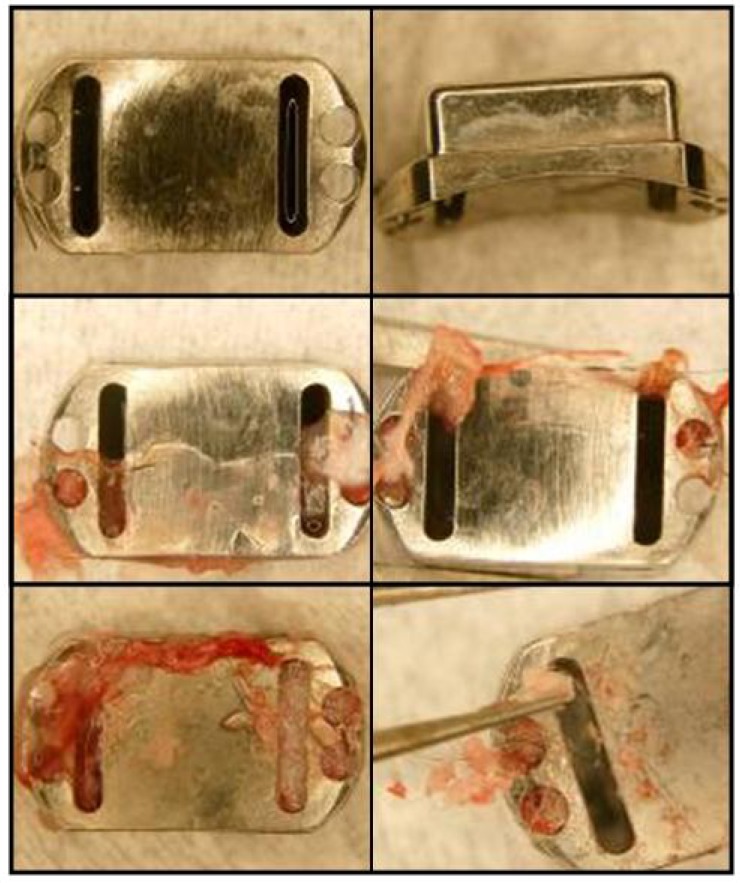
Tissue growth on Ball Bearing devices. A thin, nearly transparent tissue formed around the entire exterior of two devices (top and middle rows). These two devices had minimal to no tissue growth into the device via the “drug” ports. A third device (bottom row) fell from its implant position after 9 weeks and was filled with a white material indicative of infection.

Ball bearing devices were less likely than other devices to trigger any tissue infiltration. Of the three such devices, two showed no evidence of internal fibrous tissue, while tissue had grown in to the other through one drug port (Figure 10). This tissue growth was continuous with the outer tissue casing and had not penetrated deeper than the height of the ball bearing. 

**Figure 11:**
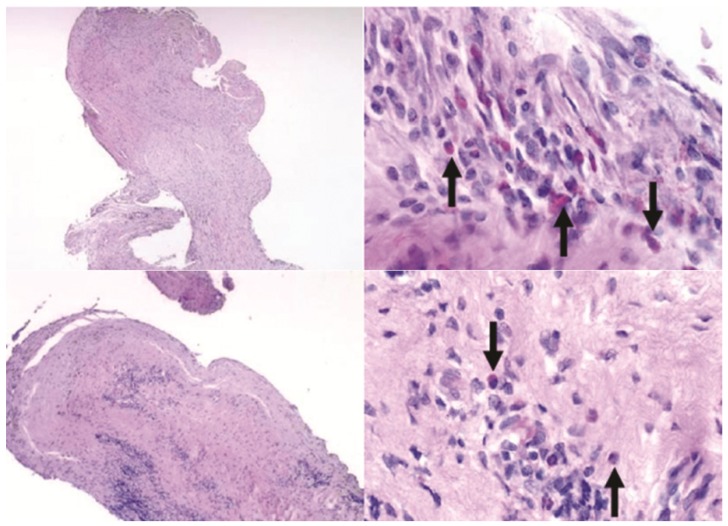
Sections of fibrous tissue residing inside devices after 10 weeks. Top row: one BB device, bottom row: one G device. Arrows indicate inflammatory cells. H&E stain. Left top: 40×, left bottom: 100×, right column: both images 600×.

Inside all devices with ingrowth, tissue was mostly fibrous with varying amounts of focal inflammation (Figure 11). There were no apparent differences in the tissue between groups, with the exception of the ball bearing device that had come out of position during the study. The latter device was filled with a highly cellular material containing a large amount of necrotic cells.

Due to functional design constraints, gearboxes had 6 openings to the outside environment and were free to move in the space under their device covers. This design allowed for a degree of microscale motion between the gearbox and its cover while on the eye. Conversely, the ball bearing device was designed as one integral unit with only 2 drug port openings. The latter device’s lower, wider profile may have limited motion of the device relative to the eye wall compared to the gear devices. The additional openings and added microscale motion in the gear devices may have contributed to greater amounts of tissue infiltrate [48]. 

These results relative to microscale motion are consistent with observations of glaucoma drainage devices (GDD’s). While draining aqueous humor may stimulate fibrous tissue formation around the device plate, both the microscale motion of the plate against the eye surface and the choice of material have been suggested as the cause of most complications [38]. Adding windows for tissue ingrowth into the plate may anchor the device, reducing the motion and decreasing the eventual fibrous capsule thickness [50]. Boswell *et al.* showed that changing to an ePTFE plate with a novel geometry encouraged a thinner, less dense fibrous capsule as compared to the standard silicone plates [42].

### 3.6. Mechanical Evaluation

Both gear and ball bearing devices required low torque to turn the moving elements before implantation, often less than 1 μNm. No statistically significant differences were observed in torque values among pre-tests at different speeds (4, 7 and 13 rpm). Pre-test torque data was averaged across motor speeds for the remainder of the analysis. Post test torque data was taken at 13 rpm.

**Figure 12:**
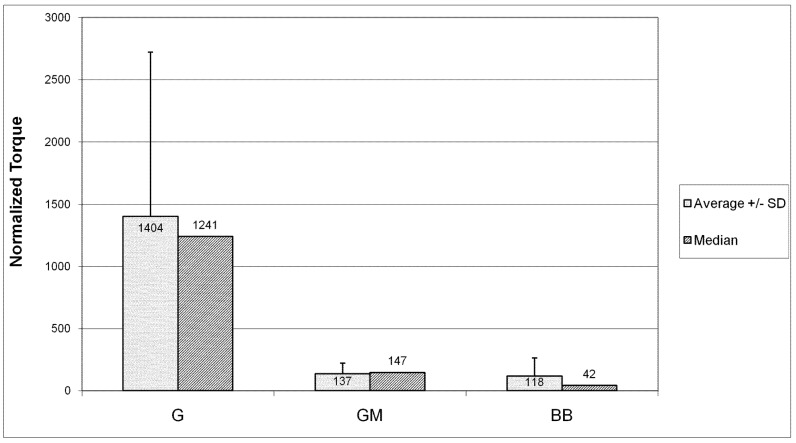
Increase in torque required to turn the moving elements. G devices were over 1000 times more difficult to turn than before 10 week implant. Adding a membrane to the base of GM devices reduced that value by an order of magnitude. BB devices showed the least increase in torque necessary to move the elements after removal from the animal.

Figure 12 shows the average (plus standard deviation) and the median of normalized torque values for each type of device. The trend in the medians shows much greater increases in torque in the G group devices compared to the other groups. Both the GM group and the BB group showed markedly smaller torque increases than the G group (p = 0.08 and p = 0.07, respectively).

Fibrous tissue infiltration appears to be the most likely cause of the deterioration in function. G devices especially showed fibrous tissue almost completely filling their interior after the 10 week residence in the body, leading to 1000 fold increases in torque requirements. Membranes covering drug delivery ports prevented communication through this opening, though fibrous tissue was still able to grow inside the device through the various other openings. In these cases, invading tissue created a sac surrounding the gears without penetrating the space between gear teeth. The organization of this tissue around the gears likely contributed to the lower torque requirements after explantation in GM devices as compared to G devices. 

The dramatic increase in power required to turn the gears presents significant challenges to device design and is likely unacceptable. The battery included in the devices comprises a significant portion of the overall volume. Additional power required to move the elements in response to scar formation would require undesirable increases in size. These results highlight the need to prevent fibrous tissue formation inside the device and the need to verify the power consumption of the fully functioning device *in vivo*. 

## 4. Conclusions

Based on this rabbit model, it can be concluded that these metal implanted devices are relatively well tolerated, especially designs presenting a low, wide profile contacting the conjunctiva and sclera. As expected with any foreign body, fibrous tissue encapsulation occurs inside and around these devices, enhanced by micro motion of the device housing, available space and disruptive geometry. For future devices of this type, it is recommended to create an integral housing design and cover any open ports in a casing. These design characteristics would be expected to reduce the rate of tissue growth into the device, therefore minimizing the disruption of device operation and increase in power consumption. With these engineering constraints, a device of this type may provide a platform for developing treatments for posterior eye diseases. 

## References

[1] Gragoudas E.S., Adamis A.P., Cunningham E.T., Feinsod M., Guyer D.R. (2004). Pegabtanib for neovascular age related macular degeneration. N. Engl. J. Med..

[2] Nowak J.Z. (2006). Age-related macular degeneration (AMD): pathogenesis and therapy. Pharmacol. Rep..

[3] Kulkarni A.D., Kuppermann B.D. (2005). Wet age-related macular degeneration. Adv. Drug Deliv. Rev..

[4] 4. Rosenfeld P.J., Brown D.M., Heier J.S., Boyer D.S., Kaiser P.K., Chung C.Y., Kim R.Y. (2006). Ranibizumab for neovascular age related macular degeneration. N. Engl. J. Med..

[5] (2006). FDA Approves New Biologic Treatment for Wet Age-Related Macular Degeneration. FDA. http://www.fda.gov/NewsEvents/Newsroom/PressAnnouncements/2006/ucm108685.htm.

[6] Ozkiris A., Erkilic K. (2005). Complications of intravitreal injection of triamcinolone acetonide. Can. J. Ophthalmol..

[7] Martidis A., Duker J.S., Greenberg P.B., Rogers A.H., Puliafito C.A., Reichel E., Baumal C. (2002). Intravitreal triamcinolone for refractory diabetic macular edema. Ophthalmology.

[8] Scott I.U., Flynn H.W. (2007). Reducing the risk of endophthalmitis following intravitreal injections. Retina.

[9] Loutsch J.M., Ong D., Hill J.M., Mitra A.K. (2003). Dendrimers. An innovative and enhanced ocular drug delivery system. Opthalmic Drug Delivery Systems. Drugs and Pharmaceutical Sciences.

[10] Ambati J., Adamis A.P. (2002). Transscleral delivery to the retina and choroid. Prog. Retin. Eye Res..

[11] Kato A., Kimura H., Okabe K., Okabe J., Kunou N., Ogura Y. (2004). Feasibility of Drug Delivery to the Posterior Pole of the Rabbit Eye with an Episcleral Implant. Invest. Ophthalmol. Vis. Sci..

[12] Okabe J., Kimura H., Kunou N., Okabe K., Kato A., Ogura Y. (2003). Biodegradable intrascleral implant for sustained intraocular delivery of betamethasone phosphate. Invest. Ophthalmol. Vis. Sci..

[13] Ghate D., Brooks W., McCarey B.E., Edelhauser H.F. (2007). Pharmacokinetics of intraocular drug delivery by periocular injections using ocular fluorophotometry. Invest. Ophthalmol. Vis. Sci..

[14] Shuler R.K., Dioguardi P.K., Henjy C., Nickerson J.M., Cruysberg L.P.J., Edelhauser H.F. (2004). Scleral permeability of a small, single-stranded oligonucleotide. J. Ocul. Pharmacol. Ther..

[15] Carrasquillo K.G., Ricker J.A., Rigas I.K., Miller J.W., Gragoudas E.S., Adamis A.P. (2003). Controlled delivery of the anti-VEGF aptamer EYE001 with poly(lactic-co-glycolic) acid microspheres. Invest. Ophthalmol. Vis. Sci..

[16] Anderson O.A., Jackson T.L., Singh J.K., Hussain A.A., Marshall J. (2008). Human transscleral albumin permeability and the effect of topographical location and donor age. Invest. Ophthalmol. Vis. Sci..

[17] Cruysberg L.P.J., Nuijts R.M.M.A., Geroski D.H., Gilbert J.A., Hendrikse F., Edelhauser H.F. (2005). The influence of intraocular pressure on the transsceral diffusion of high-molecular weight compounds. Invest. Ophthalmol. Vis. Sci..

[18] Cruysberg L.P.J., Franklin A.J., Sanders J., Self C., Yuan P., Csaky K.G., Robinson M.R., Kohn E.C., Edelhauser H.F. (2005). Effective transscleral delivery of two retinal anti-angiogenic molecules: Carboxyamido-triazole (CAI) and 2-Methoxyestradiol (2ME_2_). Retina.

[19] Ambati J., Canakis C.S., Miller J.W., Gragoudas E.S., Edwards A., Weissgold D.J., Kim I., Delori F.C., Adamis A.P. (2000). Diffusion of high molecular weight compounds through sclera. Invest. Ophthalmol. Vis. Sci..

[20] Ambati J., Gragoudas E.S., Miller J.W., You T.T., Miyamoto K., Delori F.C., Adamis A.P. (2000). Transscleral delivery of bioactive protein to the choroid and retina. Invest. Ophthalmol. Vis. Sci..

[21] Moshfeghi A.A., Peyman G.A. (2005). Micro- and nanoparticulates. Adv. Drug Deliv. Rev..

[22] Kimura H., Ogura Y. (2001). Biodegradable polymers for ocular drug delivery. Ophthalmologica.

[23] Kompella U.B., Bandi N. (2003). Ayalasomayajula SP. Subconjunctival nano- and microparticles sustain retinal delivery of budesonide, a corticosteroid capable of inhibiting VEGF experession. Invest. Ophthalmol. Vis. Sci..

[24] Barbault-Foucher S., Gref R., Russo P., Guechot J., Bochot A. (2002). Design of poly-ε-capralactone nanospheres coated with bioadhesive hyaluronic acid for ocular delivery. J. Control. Release.

[25] Vega E., Gamisans F., Garcia M.L., Chauvet A., Lacoulonche F., Egea M.A. (2008). PLGA nanospheres for the ocular delivery of flurbiprofen: drug release and interactions. J. Pharm. Sci..

[26] Eljarrat-Binstock E., Domb A.J. (2006). Iontophoresis: A non-invasive ocular drug delivery. J. Control. Release.

[27] Davies J.B., Ciavatta V.T., Boatright J.H., Nickerson J.M. (2003). Delivery of several forms of DNA, DNA-RNA hybrids, and dyes across human sclera by electrical fields. Mol. Vis..

[28] Saati S., Lo R., Li P.Y., Meng E., Varma R., Humayun M.S. (2010). Mini drug pump for ophthalmic use. Curr. Eye Res..

[29] Saati S., Lo R., Li P.Y., Meng E., Varma R., Humayun M.S. (2009). Mini drug pump for ophthalmic use. Trans. Am. Ophthalmol. Soc..

[30] Lo R., Li P.Y., Saati S., Agrawal R.N., Humayun M.S., Meng E. (2009). A passive MEMS drug delivery pump for treatment of ocular diseases. Biomed. Microdevices.

[31] Lo R., Li P.Y., Saati S., Agrawal R., Humayun M.S., Meng E. (2008). A refillable microfabricated drug delivery device for treatment of ocular diseases. Lab Chip.

[32] Borkholder D.A., Zhu X., Hyatt B.T., Archilla A.S., Livingston W.J., Frisina R.D. (2010). Murine intracochlear drug delivery: reducing concentration gradients within the cochlea. Hear. Res..

[33] Adamis A.P., Miller J.W., Gragoudas E.S., Mescher M.J., Dube C.E., Borenstein J.T., Weinstein M.G., Miller R.A., Hansberry M.L. (2009). Implantable drug delivery device and use thereof. U.S. Patent.

[34] Mescher M.J., Dube C.E., Fiering J.O., Fyler D.L., Hansberry M., Kim E.S., Borenstein J.T., Bernstein J.J., Gragoudas E., Miller J. (2006). A programmable device for long-term transscleral drug delivery. Transactions of the 31st annual meeting of the Society for Biomaterials, Pittsburgh, PA, USA.

[35] Dee K.C., Puleo D.A., Bizios R. (2002). An Introduction to Tissue-Biomaterial Interactions.

[36] Ratner B.D. (2002). Reducing capsular thickness and enhancing angiogenesis around implant drug release systems. J. Control. Release.

[37] Anderson J.M., Langone J.J. (1999). Issues and perspectives on the biocompatibility and immunotoxicity evaluation of implanted controlled release systems. J. Control. Release.

[38] Lloyd A.W., Faragher R.G., Denyer S.P. (2001). Ocular biomaterials and implants. Biomaterials.

[39] D’Hermies F., Korobelnik J-F., Caputo G., Mashhour B., Chauvaud D., Pouliguen Y., Renard G. (1998). Encapsulation of scleral buckling materials: A study of six specimens. Ophthalmology.

[40] Ayyala R.S., Harman L.E., Michelini-Norris B., Ondrovic L.E., Haller E., Margo C.E., Stevens S.X. (1999). Comparison of different biomaterials for glaucoma drainage devices. Arch. Ophthal..

[41] Cohen S.J. (2007). Biocompatibility of an Implantable Ophthalmic Drug Delivery Device. MS Thesis.

[42] Boswell C.A., Noecker R.J., Mac M., Snyder R.W., Williams S.K. (1999). Evaluation of an aqueous drainage glaucoma device constructed of ePTFE. J. Biomed. Mater. Res..

[43] Voskerician G., Shive M.S., Shawgo R.S., von Recum H., Anderson J.M., Cima M.J., Langer R. (2003). Biocompatibility and biofouling of MEMS drug delivery devices. Biomaterials.

[44] Dasgupta G., BenMohamed L. (2011). Of mice and not humans: How reliable are animal models for evaluation of herpes CD8+-t cell-epitopes-based immunotherapeutic vaccine candidates?. Vaccine.

[45] Liang H., Baudouin C., Dupas B., Brignole-Baudouin F. (2010). Live conjunctiva-associated lymphoid tissue analysis in rabbit under inflammatory stimuli using *in vivo* confocal microscopy. IOVS.

[46] Stephen A.E., Masiakos P.T., Segev D.L., Vacanti J.P., Donahoe P.K., MacLaughlin D.T. (2001). Tissue engineered cells producing complex recombinant proteins inhibit ovarian cancer *in vivo*. Proc. Natl. Acad. Sci. USA.

[47] Shin M., Yoshimoto H., Vacanti J.P. (2004). *In vivo* tissue engineering using mesenchymal stem cells on a novel electrospun nanofibrous scaffold. Tissue Eng..

[48] Spector M., Lalor P.A., Ratner B.D., Hoffman A.S., Schoen F.J., Lemons J.E. (1996). *In vivo* assessment of tissue compatibility. Biomaterials Science, An Introduction to Materials.

[49] Sanders J.E., Rochefort J.R. (2003). Fibrous encapsulation of single polymer microfibers depends on their vertical dimension in subcutaneous tissue. J. Biomed. Mater. Res. A.

[50] Ayyala R.S., Duarte J.L., Sahiner N. (2006). Glaucoma drainage devices: state of the art. Expert Rev. Med. Devices.

